# Multiple lung abscesses and cold agglutinin syndrome following coronavirus disease 2019: a case report

**DOI:** 10.1186/s13256-024-04648-3

**Published:** 2024-07-13

**Authors:** Masaharu Aga, So Okubo, Toshiki Ikeda, Yuko Higashi, Yusuke Hamakawa, Suguru Matsuzaka, Kazuhito Miyazaki, Yuri Taniguchi, Yuki Misumi, Yoko Agemi, Yukiko Nakamura, Tsuneo Shimokawa, Yoshinobu Aisa, Hiroaki Okamoto

**Affiliations:** 1 Department of Respiratory Medicine, Yokohama Municipal Citizen’s Hospital, 1-1, Mitsuzawa Nishi Machi, Kanagawa Ku, Yokohama Shi, Kanagawa Ken 221-0855 Japan; 2 Department of Hematology, Yokohama Municipal Citizen’s Hospital, Yokohama, Kanagawa Japan; 3 Department of Transitional and Palliative Care, Iizuka Hospital, Iizuka, Japan

**Keywords:** Coronavirus disease 2019, Lung abscess, Secondary cold agglutinin syndrome

## Abstract

**Background:**

Cold agglutination syndrome is a subtype of autoimmune hemolytic anemia. The condition is referred to as “cold” because the antibodies become active and induce hemolysis at cold temperatures, typically 3–4 °C, which is not always the case in other kinds of autoimmune hemolytic anemia. Whereas primary cold agglutination syndrome may occur in the absence of underlying conditions, secondary cold agglutination syndrome is associated with the presence of underlying infections, including coronavirus disease 2019.

**Case presentation:**

We report the case of a 69-year-old Japanese woman with periodontitis who was referred to our hospital with complaints of brown-colored urine and chest pain. Her hemoglobin level was 6.1 g/dL. Computed tomography revealed multiple lung abscesses. Her direct antibody test results were positive (2+) for anti-complement direct antiglobulin and negative for immunoglobulin G, and her cold agglutinin titer was elevated at 1:4096. Workup for anemia revealed a positive result for cold agglutination syndrome. The patient had received the fourth dose of coronavirus disease 2019 vaccination. Nasopharyngeal swab test for detecting severe acute respiratory syndrome coronavirus 2 using a real-time reverse-transcription polymerase chain reaction gave a cycle threshold value of 42.3, and the level of virus-specific immunoglobulin G was elevated at 7.71 S/C (normal range −1.4 S/C).

**Conclusion:**

A decrease in hemoglobin in patients with coronavirus disease 2019 may be associated with secondary cold agglutination syndrome. The patient was hypothesized to have developed multiple lung abscesses with secondary cold agglutination syndrome following coronavirus disease 2019. Thus, following coronavirus disease 2019, patients can develop secondary cold agglutination syndrome, which could worsen owing to associated bloodstream bacterial infections.

## Background

Cold agglutination syndrome (CAS) is a form of autoimmune hemolytic anemia (AIHA). This condition is characterized by the presence of autoantibodies, known as cold agglutinins, which cause agglutination, with an optimum temperature of 3–4 °C, when the red blood cells circulate in cooler parts of the body [1]. Primary CAS may occur in the absence of underlying conditions. In contrast, secondary CAS is associated with the presence of underlying infections such as *Mycoplasma pneumoniae* pneumonia and Epstein–Barr virus infection, autoimmune disorders, and lymphoid malignancies [2]. Severe acute respiratory syndrome coronavirus 2 (SARS-CoV-2), the viral agent responsible for coronavirus disease 2019 (COVID-19), can induce many hematological abnormalities. Furthermore, several case reports and case series have suggested that AIHA, including secondary CAS, may be associated with COVID-19 [3–6]. Herein, we report a case of multiple lung abscesses with secondary CAS following COVID-19.

## Case presentation

A 69-year-old Japanese woman with hypertension, dyslipidemia, and periodontitis under dental care complained of brown-colored urine and chest pain. She had visited her primary care physician 5 days prior to visiting our hospital and was prescribed amoxicillin, which did not improve her symptoms. She was referred to our hospital with elevated bilirubin levels and multiple lung mass shadows observed on computed tomography (CT). The patient had no history of smoking or alcohol consumption. She had received the fourth dose of COVID-19 vaccination, with the Pfizer-BioNTech BNT16B2b2 mRNA vaccine, 7 months prior to presentation. The patient’s temperature was 36.7 °C, heart rate was 98 beats per minute, blood pressure was 115/75 mmHg, respiratory rate was 24 breaths per minute, and oxygen saturation was 90% on ambient air. On physical examination, there was no evidence of hepatomegaly or lymphadenopathy; however, reduced bilateral breath sounds and jaundice throughout the patient’s body were noted.

**Fig. 1:**
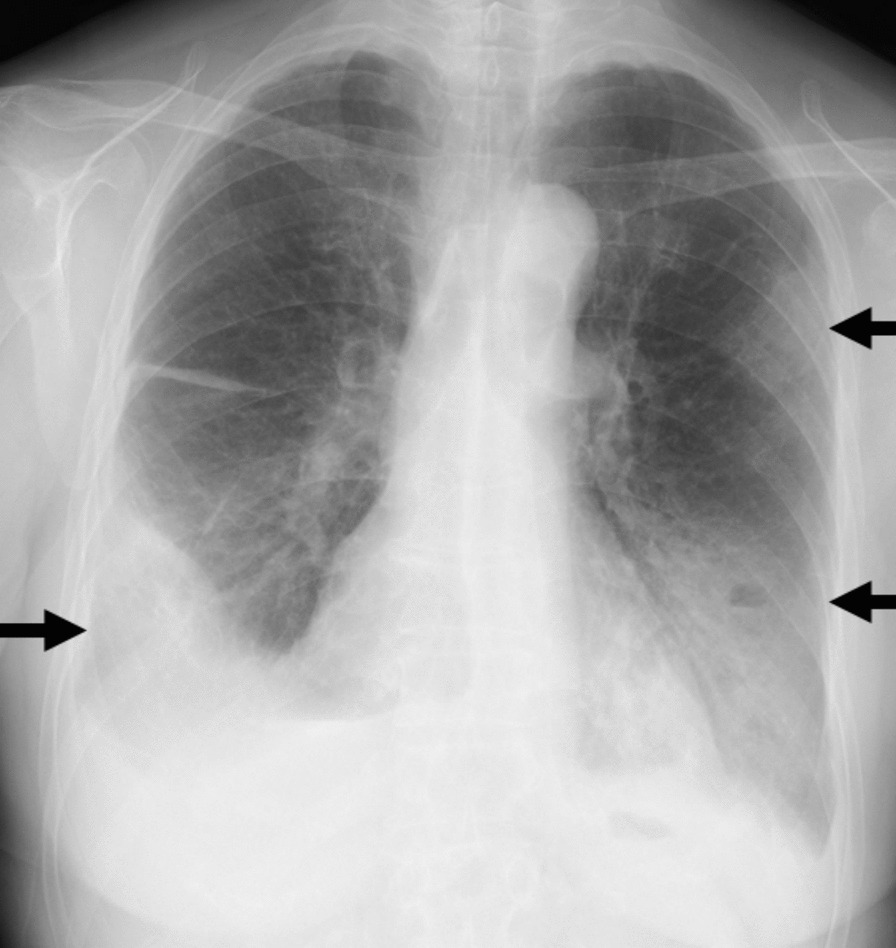
Chest radiograph at presentation revealing bilateral consolidation with an air–fluid level (black arrows)

**Fig. 2:**
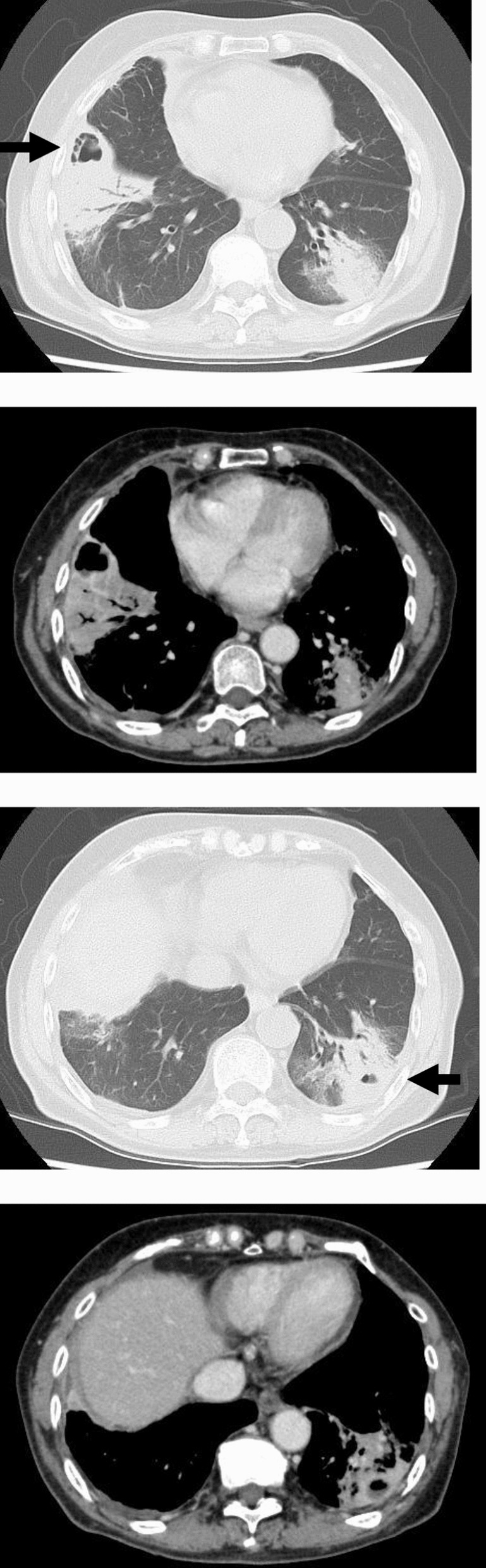
Computed tomography at presentation showing multiple lung abscesses (black arrows)

Laboratory tests at admission revealed a white blood cell count of 20.3 × 10^3^ cells/μL, hemoglobin (Hb) level of 6.1 g/dL, and platelet count of 519 × 10^3^/μL. Other biochemical tests showed an elevation of bilirubin (total bilirubin, 6.1 mg/dL; direct bilirubin, 1.5 mg/dL) and C-reactive protein (31.7 mg/dL) (laboratory data are summarized in Table 1). The workup for acute anemia revealed serum iron levels of 135 μg/dL (normal range 40–188 μg/dL), ferritin level of 6,218 μg/dL (normal range 4.63–204 μg/dL), and iron saturation of 91.8% (normal range 20–55%). The reticulocyte index was 1.9% (normal range 0.5–2.5%). The lactate dehydrogenase level was 445 U/L (normal range 124–222 U/L), and the D-dimer concentration was 8.94 μg/mL (normal range < 1.0 μg/mL). Haptoglobin levels had decreased beyond the detection sensitivity. Her direct antibody test results were positive (2+) for anti-complement direct antiglobulin and negative for immunoglobulin (Ig)G, and her cold agglutinin titer was elevated at 1:4096. Serum protein electrophoresis did not detect monoclonal gammopathy, including IgM, and flow cytometry of the peripheral blood did not show B-cell clonality. Transthoracic echocardiography revealed no evidence of valvular disease or vegetation. Chest radiography revealed bilateral consolidation with an air–fluid level (Fig. 1). Whole-body CT revealed multiple masses or nodule consolidation at the air–fluid interface of the lung and no signs of active bleeding, thrombosis, hepatomegaly, or lymphadenopathy (Fig. 2). A nasopharyngeal swab test for detecting SARS-CoV-2 using real-time reverse-transcription polymerase chain reaction (RT-PCR) yielded a cycle threshold (Ct) value of 42.3, and the level of virus-specific immunoglobulin G (IgG) was elevated at 7.71 S/C (normal range < 1.4 S/C).

**Table 1:** Laboratory data during the course of the disease

Variable	Reference range (adult)	Day 1	Day 2	Day 4	Day 7	Day 14	Week 4	Week 8
Hemoglobin (g/dL)	11.6–14.8	6.1	4.4	6.4	7.5	8.5	10.6	12.7
Hematocrit (%)	35.1–44.4	18.3	13.5	18.5	22.8	26.7	34.2	40.4
White blood cell count (× 10^3^/μL)	3.3–8.6	20.3	17	23.2	14.9	5.9	5.6	6.1
Platelet count (× 10^3^/μL)	158–348	519	540	494	641	1085	462	311
RBC (10^6^/μL)	3.86–4.92	2.02	1.5	2.11	2.46	2.71	3.39	4.17
MCV (fL)	83.6–89.2	90.6	90	87.7	92.7	98.5	100.9	96.9
ALP (U/L)	38–113	172	148	127	106	105	126	107
Bilirubin (mg/dL)								
Total	0.4–1.5	6.1	4.1	2.2	0.8	0.5	0.5	0.4
Direct	0.0–0.5	1.5	1.1	0.5	0.2	0.1	0.1	0
AST (U/L)	13–30	34	30	28	41	24	18	18
ALT (U/L)	7–23	62	45	36	60	47	20	19
LDH (U/L)	124–222	445	486	384	291	179	187	179
C-reactive protein (mg/dL)	< 0.14	31.7	28.3	17.4	8.9	1.7	0.3	0
Cold agglutinin titers	< 256		4096				1024	256

*RBC* red blood cell, *MCV* mean corpuscular volume, *ALP* alkaline phosphatase, *AST* aspartate aminotransferase, *ALT* alanine transaminase, *LDH* lactate dehydrogenase

Antibacterial treatment with piperacillin/tazobactam (4.5 g, three times a day) was started. She did not receive any specific treatment for COVID-19 because she was thought to have contracted the disease some time ago based on the high Ct value of the RT-PCR test and elevated IgG for SARS-CoV-2-specific IgG. On the second day of admission, her Hb level dropped to 4.4 g/dL, and she was transfused with 4 units of blood. On the third day of admission, chest radiography revealed bilateral pleural effusions. The thoracentesis fluid showed an exudative pleural effusion with neutrophil predominance; however, chest drainage could not be performed because the amount of pleural effusion was small. Sputum cultures revealed normal flora, and thoracentesis fluid and blood cultures revealed no pathogens. The patient improved clinically, her Hb level stabilized to 9.0 g/dL during the 3 weeks of hospital stay, and cold agglutinin titers gradually decreased. Eight weeks after presentation, cold agglutinin titers normalized, and chest radiography showed only slight shadows. There were no treatment-related adverse effects.

## Discussion

We report the first case of multiple lung abscesses complicated with secondary CAS following COVID-19. This case demonstrates two crucial issues. First, patients can develop secondary CAS, which could worsen owing to bacterial infections after COVID-19. Second, COVID-19, even without hospitalization, might increase the risk of bloodstream infections.

Although the precise mechanism by which COVID-19 induces CAS is yet to be determined, molecular mimicry has been considered to account for the induction of SARS-CoV-2-induced autoimmune phenomena, including AIHA [7]. A systematic review conducted in 2021 reported that 50 patients were diagnosed with AIHA, including CAS, secondary to SARS-CoV-2 infection or vaccination [3]. Our patient presented with multiple lung abscesses simultaneously with secondary CAS, with no radiographic evidence of COVID-19 pneumonia. Since there have been no reports of secondary CAS being triggered by lung abscesses alone, we could not explain the elevated cold agglutinin levels caused by the lung abscesses. However, the secondary CAS and lung abscesses in this patient improved spontaneously without any COVID-19-specific treatment. Therefore, although COVID-19 is thought to play a central role, bacterial infection might be an exacerbating factor.

Lung abscesses are classified as primary or secondary depending on the etiology [8]. Primary lung abscesses result from aspiration of oropharyngeal secretions, and secondary lung abscesses occur when there is a predisposing condition such as bronchial obstruction, hematogenous spread, or an immunocompromised status. Several studies have reported that periodontitis causes septic pulmonary embolism, which could lead to secondary lung abscesses [9, 10]. Blood, thoracentesis fluid, and sputum cultures showed no pathogens; therefore, whether her radiographic abnormalities were induced by bacterial infection was unknown. However, it was likely that bloodstream infections from periodontitis caused secondary lung abscesses in this patient because there were multiple shadows, the distribution of the shadows was peripheral dominant, thoracentesis was neutrophil predominant, and her condition improved clinically only with antibiotics. Only 8.1% of periodontal disease-associated septic pulmonary embolism cases have reported positive blood culture [10]. A systematic review revealed that the rate of occurrence of bloodstream infections was 7.3% [95% confidence interval (CI) 4.7–11.0%] in hospitalized patients with COVID-19 [11], which is higher than that for patients without COVID-19 (odds ratio 2.77; 95% CI 1.53–5.02). The present case suggests that COVID-19 might be related to bloodstream infections even without hospitalization. Lung abscesses as a complication of COVID-19 have been reported earlier in nonintubated COVID-19 cases (12, 13). However, in each of these cases, only a single cavitary lesion was found. Hence, bloodstream infection due to periodontitis was more likely to be the suspected etiology for this case.

## Conclusions

This case report describes a patient who developed multiple lung abscesses with secondary CAS following COVID-19. The patient’s Hb level stabilized, and cold agglutinin titers slowly decreased with clinical improvement of the lung abscesses without COVID-19 treatment. The abrupt drop in Hb levels observed in patients with COVID-19 may be associated with secondary CAS, and clinicians should remain alert for such complications. This is the first report of lung abscess complicated with secondary CAS after COVID-19. However, there is a need for continuous observation to evaluate whether COVID-19, even when it does not require hospitalization, is related to the development of secondary infections. In addition, more case reports are needed to establish the association of lung abscess with secondary CAS.

## Data Availability

Not applicable.

## Abbreviations

AIHA: Autoimmune hemolytic anemia
CAS: Cold agglutination syndrome
COVID-19: Coronavirus disease 2019
CT: Computed tomography
RT-PCR: Real-time reverse-transcription polymerase chain reaction.
SARS-CoV-2: Severe acute respiratory syndrome coronavirus 2

## References

[1] Berentsen S, Tjønnfjord GE (2012). Diagnosis and treatment of cold agglutinin mediated autoimmune hemolytic anemia. Blood Rev.

[2] Gertz MA (2005). Cold agglutinin disease and cryoglobulinemia. Clin Lymphoma.

[3] Jacobs JW, Booth GS (2022). COVID-19 and immune-mediated RBC destruction. Am J Clin Pathol.

[4] Tsukamoto Y, Umeda M, Muto Y, Sugimoto T, Yamauchi M, Ando K (2022). Severe anemia due to cold agglutinin syndrome in a COVID-19 patient with IgM monoclonal gammopathy of undetermined significance successfully treated with corticosteroids. Intern Med.

[5] Kaur J, Mogulla S, Khan R, Krishnamoorthy G, Garg S (2021). Transient cold agglutinins in a patient with COVID-19. Cureus.

[6] Patil NR, Herc ES, Girgis M (2022). Cold agglutinin disease and autoimmune hemolytic anemia with pulmonary embolism as a presentation of COVID-19 infection. Hematol Oncol Stem Cell Ther.

[7] Damoiseaux J, Dotan A, Fritzler MJ, Bogdanos DP, Meroni PL, Roggenbuck D (2022). Autoantibodies and SARS-CoV2 infection: the spectrum from association to clinical implication: report of the 15th Dresden symposium on autoantibodies. Autoimmun Rev.

[8] Kuhajda I, Zarogoulidis K, Tsirgogianni K, Tsavlis D, Kioumis I, Kosmidis C (2015). Lung abscess-etiology, diagnostic and treatment options. Ann Transl Med.

[9] Shiota Y, Arikita H, Horita N, Hiyama J, Ono T, Ohkawa S (2002). Septic pulmonary embolism associated with periodontal disease: reports of two cases and review of the literature. Chest.

[10] Watanabe T, Yokoe M, Noguchi Y (2019). Septic pulmonary embolism associated with periodontal disease: a case report and literature review. BMC Infect Dis.

[11] Ippolito M, Simone B, Filisina C, Catalanotto FR, Catalisano G, Marino C (2021). Bloodstream infections in hospitalized patients with COVID-19: a systematic review and meta-analysis. Microorganisms.

[12] Zamani N, Aloosh O, Ahsant S, Yassin Z, Abkhoo A, Riahi T (2021). Lung abscess as a complication of COVID-19 infection, a case report. Clin Case Rep.

[13] Umamoto K, Horiba M (2023). Lung abscess as a secondary infection of COVID-19: a case report and literature review. J Infect Chemother.

